# Cinnamaldehyde nanoemulsion decorated with rhamnolipid for inhibition of methicillin-resistant *Staphylococcus aureus* biofilm formation: *in vitro* and *in vivo* assessment

**DOI:** 10.3389/fmicb.2024.1514659

**Published:** 2024-12-24

**Authors:** Lizi Yin, Yingzi Guo, Xiyuan Xv, Yuyun Dai, Luxin Li, Fengsheng Sun, Xue Lv, Gang Shu, Xiaoxia Liang, Changliang He, Zhiwen Xu, Ping Ouyang

**Affiliations:** College of Veterinary Medicine, Sichuan Agricultural University, Wenjiang, China

**Keywords:** nanoemulsion, biofilm, cinnamaldehyde, rhamnolipid, MRSA

## Abstract

**Background:**

*Staphylococcus aureus* (*S. aureus*) biofilm associated infections are prevalent and persistent, posing a serious threat to human health and causing significant economic losses in animal husbandry. Nanoemulsions demonstrate significant potential in the treatment of bacterial biofilm associated infections due to their unique physical, chemical and biological properties. In this study, a novel cinnamaldehyde nanoemulsion with the ability to penetrate biofilm structures and eliminate biofilms was developed.

**Methods:**

The formulation of cinnamaldehyde nanoemulsion (Cin-NE) combined with rhamnolipid (RHL) was developed by self-assembly, and the efficacies of this formulation in inhibiting *S. aureus* biofilm associated infections were assessed through *in vitro* assays and *in vivo* experiments by a mouse skin wound healing model.

**Results:**

The particle size of the selected Cin-NE formulation was 13.66 ± 0.08 nm, and the Cin-RHL-NE formulation was 20.45 ± 0.25 nm. The selected Cin-RHL-NE formulation was stable at 4, 25, and 37°C. Furthermore, the Minimum Inhibitory Concentration (MIC) value of Cin-RHL-NE against MRSA was two-fold lower than drug solution. Confocal laser scanning microscopy (CLSM) revealed the superior efficacy of Cin-RHL-NE in eradicating MRSA biofilms while maintaining the Cin’s inherent functional properties. The efficacy of Cin-RHL-NE in the mouse skin wound healing model was superior to other formulation.

**Conclusion:**

These findings highlight the potential of the formulation Cin-RHL-NE for eradicating biofilms, and effective in treating notoriously persistent bacterial infections. The Cin-RHL-NE can used as a dosage form of Cin application to bacterial biofilm associated infections.

## 1 Introduction

Methicillin-resistant *Staphylococcus aureus* (MRSA) is frequently encountered in healthcare facilities and communities on a global scale, presenting a significant threat to public health (Chambers and Deleo, 2009; DeLeo et al., 2010). Bacterial biofilms serve as a sophisticated defense mechanism, offering bacteria protection from antimicrobials and the host immune system through their phenotypic resistance. In the treatment of MRSA infections, dealing with biofilms presents a particularly formidable challenge (Nelson, 2003). The sustained emergence and rapid spread of MRSA biofilm-associated infections, coupled with the lack of new antibiotics and the ability of drugs to penetrate biofilm structures to eradicate them, the urgent need for novel antimicrobial agents and innovative therapeutic strategies (Jamal et al., 2018). In response to this crisis, numerous nations and territories have implemented prohibitions on the utilization of antibiotics as supplements in animal feed, encouraging the exploration and development of alternative molecules, particularly those sourced from natural origins (Singhai et al., 2012). In this context, natural products could play a crucial role due to their antimicrobial and anti-biofilms effects (Cruz et al., 2018). For example, certain essential oils have been shown to effectively inhibiting biofilms formed by *Pseudomonas aeruginosa*, *Pseudomonas putida*, and *S. aureus* (Kavanaugh and Ribbeck, 2012).

Cinnamaldehyde (Cin), a naturally occurring hydrophobic aromatic aldehyde that is abundantly present in Cinnamomum plants, particularly in *C. cassia (L.) D. Don.*, is widely used in food and animal husbandry industries. Numerous pharmacological studies have demonstrated that Cin is a broad-spectrum antimicrobial and anti-biofilms agent with significant effects against strains of *S. aureus*, *Escherichia coli*, *Salmonella* spp., *Pseudomonas aeruginosa* and *Bacillus* spp. (Chalchat and Valade, 2000). Cinnamaldehyde has also been shown to be effective against biofilms formed by both Gram-positive and Gram-negative bacteria, such as *Pseudomonas aeruginosa* and *Staphylococcus aureus* (Topa et al., 2018; Zhang et al., 2014). However, the clinical application of Cin is limited due to its unique physicochemical property (instability, water insoluble, high volatility, and irritating odor) and lack of biofilm targeting.

Various carrier systems, including liposomes, nanoparticles, and nanoemulsions (NEs) (Chen et al., 2022; Feng et al., 2023; Qin et al., 2024), have been developed to effectively enhance the solubility, dissolution rate, and bioavailability of these components. Gene editing technologies for biofilm eradication are also available, such as CRISPRi, a technology to inhibit a specific gene in drug-resistant bacteria, which affects bacterial adhesion and biofilm formation (Zuberi et al., 2017a; Zuberi et al., 2022; Zuberi et al., 2017b). However, these methods have specific sequence requirements, are technically complex, and carry a risk of off-target effects (Zuberi et al., 2017a; Zuberi et al., 2022; Zuberi et al., 2017b). While previous studies have investigated the formulations of Cin-NE and their antimicrobial potential (Yuan Gao, 2021), a significant challenge in clinical practice remains: managing biofilm-associated infections caused by drug-resistant bacteria, such as MRSA. The key to overcoming these infections lies in effectively eradicating biofilms; however, this critical aspect has not been sufficiently addressed by existing research.

Rhamnolipid (RHL) is a biosurfactant that offers enhanced surface and interfacial activity, lower biotoxicity, and easier degradation compared to conventional emulsifiers (Mohanty and Mukherji, 2013). Previous studies have shown that RHL can disrupt the extracellular matrix of *Staphylococcus aureus* biofilm cells, making it easier for antimicrobial drugs to penetrate into the biofilm and kill the bacteria within (Thakur et al., 2021).

In the current study, a novel formulation of Cin-NE fortified with RHL was developed to enhance solubility and biofilm eradication capabilities. An optimal nanoemulsion preparation was established using Central Composite Design-Response Surface Methodology (CCD-RSM). The CCD-RSM has the merits that it can effectively optimize the effects of multiple variables within a limited number of experiments, thereby saving time and resources. This approach aids in more accurately assessing the impact of different factors on the outcomes and is easy of implementation (Bhattacharya, 2021). The efficacy of this formulation in inhibiting MRSA proliferation was evaluated through both *in vitro* assays and *in vivo* experiments using a mouse skin wound healing model, comprehensively assessing its therapeutic potential.

This work provides new insights and a theoretical basis for the production and application of Cin as a natural, large-scale antibacterial agent, particularly against MRSA biofilms, thereby advancing the frontier of strategies for managing resistant biofilms.

## 2 Materials and methods

### 2.1 Materials

The MRSA strain USA300 (ATCC®BAA-1717TM) [obtained from the American Type Culture Collection (ATCC)] was used in the current study and was cultivated in brain heart infusion (BHI) broth (Hopebio, Qingdao, China). Cinnamaldehyde (>98% HPLC purity, Shanghai McLin Biochemical Technology Co., Ltd.) and dissolved in dimethyl sulfoxide to obtain a stock solution, Rhamnolipid (Shanghai McLin Biochemical Technology Co., Ltd.), Tween 80 (Chengdu Kolon Chemicals Ltd.), Ethanol (Chengdu Kolon Chemicals Ltd.), Polyethylene Glycol 400 (PEG 400, Chengdu Kolon Chemicals Ltd.), Cremophor EL (EL-40, Chengdu Kolon Chemicals Ltd.), Ethyl Acetate (Chengdu Kolon Chemicals Ltd.) and distilled water (Chengdu Kolon Chemicals Ltd). All chemical reagents were of analytical purity and were used directly without further purification.

### 2.2 Preparation of nanoemulsions

#### 2.2.1 Preparation method of nanoemulsion

Cinnamaldehyde nanoemulsion (Cin-NE) was prepared using the spontaneous emulsification method, which overcomes the disadvantages of high-energy emulsification, such as high initial equipment and operating costs, high power requirements, potential for equipment breakdown, and difficulties in producing very fine droplets (Ying Yang, 2012). First, 0.15 g of Cin was added to 0.28 g of ethyl acetate and stirred at 800 rpm with a magnetic stirrer (Tianjin SAIDELISI Ltd.). Next, 1.174 g of EL-40 and 0.546 g of ethanol were added to the mixture, followed by the gradual addition of 0.85 g of distilled water while continuing to stir until a transparent, uniform yellow Cin-NE system was formed. The preparation of Cin-RHL-NE followed the same procedure as Cin-NE, with the exception that 0.075 g of Rhamnolipid (RHL) was added with the addition of 1.174 g of EL-40 and 0.546 g of ethanol, while all other steps remained identical.” The preparation of Blank-RHL-NE (RHL-Blank-NE) was similar to that of Cin-RHL-NE, with the only difference being the absence of Cin. The preparation of Blank-NE was similar to that of Cin-NE, with the only difference being the absence of Cin.

#### 2.2.2 Screening of Cin-NE oil phase

Previous laboratory research found that ethyl acetate has good solubility for cinnamaldehyde. 5 mL of cinnamaldehyde was add to 3 mL of ethyl acetate, mixed well. Then it was centrifuged at 10000 r/min for 15 min and the state of the mixture was observed visually.

#### 2.2.3 Screening of Cin-NE emulsifier, co-emulsifier and Km value (emulsifier: co-emulsifier)

For the NE formulation, two emulsifiers—Tween 80 and EL-40—were screened. The emulsifier for the nanoemulsion drug delivery system was determined using water titration combined with the pseudo-ternary phase diagram method. Initially, ethanol was chosen as the co-emulsifier, with ethyl acetate designated as the oil phase component. The emulsifier was mixed with the co-emulsifier at a mass ratio of 2:1, and then the mixed emulsifier was combined with the oil phase at mass ratios of 9:1, 8:2, 7:3, 6:4, 5:5, 4:6, 3:7, 2:8, and 1:9. Distilled water was then added drop-by-drop under stirring conditions of 800 r/min at 25(±1)°C. The critical volume of water that led to emulsion destabilization was recorded. Origin 2019 software was used to plot the pseudo-ternary phase diagram and observe the emulsion-forming region. The extent of the nanoemulsion region on the pseudo-ternary phase diagram was used as an indicator to determine the more suitable emulsifier (Liu et al., 2022).

For the co-emulsifier screening, ethanol and PEG 400 were evaluated. The selected emulsifier was combined with each co-emulsifier at a ratio of 2:1, forming a mixed emulsifier. This mixed emulsifier was then blended with ethyl acetate. Water titration was performed as described earlier, and the pseudo-ternary phase diagram was plotted using Origin 2019 to observe the emulsion-forming region. The extent of the nanoemulsion area on the pseudo-ternary phase diagram was used as the criterion to determine the more appropriate co-emulsifier.

The oil phase, emulsifier, and co-emulsifier components have been determined in the aforementioned screening. The fixed oil phase was ethyl acetate, the emulsifier was EL-40, and the co-emulsifier was ethanol. Subsequently, further screening of the ratio between the emulsifier and co-emulsifier was conducted. The emulsifier and co-emulsifier were mixed in ratios of 1:1, 2:1, 3:1, 1:2, and 1:3. Using the water titration method, the Km value (emulsifier: co-emulsifier) was determined from the size of the nanoemulsion region area of the pseudo-ternary phase diagram.

#### 2.2.4 Prescription optimization

Central Composite Design Response Surface Methodology (CCD-RSM) was employed to investigate the impact of two independent variables, X1 (oil phase) and X2 (Km), on the dependent response variables and to predict the optimal response values (Anjum et al., 2024). The oil phase used was ethyl acetate, and the Km (EL-40: ethanol) ratio ranged from 1 to 3. The influence of these parameters was assessed with respect to two critical response variables: Y1, particle size (nm), and Y2, maximum drug-loading capacity (g/mL). Determine the particle size by measuring the Blank-NE configuration of X1 (oil phase) and X2 (Km) fitted by CCD-RSM using a particle size analyzer (Delsa NanoC, Beckman Coulter, USA). Cin was add to the Blank NE configured by CCD-RSM fitting, and record the amount of added Cin when the Blank-NE becomes turbid to determine the maximum drug-loading capacity. The experimental design and statistical analysis were conducted using Design-Expert® version 13 software. The factors of the oil phase and Km were set at five levels (*α*-, 1-, 0, 1 +, α +) to evaluate their effects on the response variables, as summarized in Table 1. This setup, which involved the interaction of two factors across five levels with six replicates at the central point, resulted in a randomized scheme for conducting 20 experiments. The optimal formulation was determined by integrating the objectives of minimizing mean particle size and maximizing drug-loading capacity, as guided by the contour plots of the response surface. The predicted optimal formulation was then validated through relevant experimental procedures.

**Table 1:** CCD-RSM factors of the oil phase and Km.

Factor	Level				
	−α	−1	0	+1	+α
Oil phase (X1)	10	14.3934	25	35.6066	40
Km (X2)	1	1.29289	2	2.70711	3

#### 2.2.5 Rhamnolipid dosage screening

The preparation method of the Cin-RHL-NE was the same as described above. Cin and RHL were screened at ratios of 1:1, 2:1, 3:1, and 4:1. The amount of rhamnolipid added was determined by particle size and PDI (Polydispersity Index).

### 2.3 Characterization of Cin-NE, Cin-RHL-NE

#### 2.3.1 Emulsion type identification

5 mL samples of Cin-NE, Cin-RHL-NE, Cin emulsion, and distilled water were dispensed into vials at 25°C and divided into four groups: Cin-NE (bottle 1), Cin-RHL-NE (bottle 2), Cin emulsion (bottle 3), and negative control (bottle 4). Cin-NE and Cin-RHL-NE were prepared using the method described in the “Preparation of Nanoemulsions” section. Add excess Cin (0.3 g/mL) to Blank-NE to prepare Cin emulsion. The visual appearance, Tyndall effect, diffusion rate of the water-soluble dye (methylene blue), and the oil-soluble dye (Sudan Red III) were then assessed (He et al., 2017).

#### 2.3.2 Particle size measurement and morphological characterization

Cin-NE and Cin-RHL-NE were prepared, and their particle size, PDI, and zeta potential were measured at 25°C using a laser particle size/potential analyzer (Delsa NanoC, Beckman Coulter, USA) (Lei et al., 2019). These measurements were replicated in triplicate. The micro-morphology of the nanoparticles was characterized and observed using a transmission electron microscope (JEM2100, Nippon Electron Co., Ltd., Japan) (Khan et al., 2015).

#### 2.3.3 Encapsulation efficiency of Cin-NE

A standard curve was established via UV spectrophotometry (UV-2600i, Shimadzu, Japan) for the determination of Cin content. Take 500 μL Cin-NE and centrifuge it in an ultrafiltration tube (1,000 KD) at 10000 r/min for 15 min to obtain the clarified centrifugal solution to measure the content of total active ingredient (W_t_) and concentration of free active substance (W_f_). Absorbance was measured using the UV spectrophotometer at OD_284_. Encapsulation efficiency (EE%) were calculated from the total and free concentrations (Feng et al., 2022).

$\mathrm{Encapsulation efficiency}\;\left(EE\%\right)=\frac{\left(Wt-Wf\right)}{Wt}\times 100\%$ .

#### 2.3.4 Stabilities of Cin-NE and Cin-RHL-NE

To verify the stability of the formulation under different environmental conditions, stability tests were conducted. The selected Cin-NE and Cin-RHL-NE underwent various tests, including centrifugation (6,000 r/min, 8,000 r/min, 10,000 r/min, each lasting 10 min), storage at different temperatures (−20°C, 4°C, 25°C), pH variations (1.00, 4.00, 7.00, 9.18), and exposure to light (0 Lx, 400 Lx) (Li et al., 2018). The particle size of nanoemulsions after different treatments was measured according to the method in 2.3.2.

### 2.4 Evaluation method for antimicrobial activity *in vitro*

#### 2.4.1 Determination of NEs antimicrobial activity on MRSA planktonic bacteria

##### 2.4.1.1 Determination of MIC and MBC

The minimum inhibitory concentration (MIC) and the minimum bactericidal concentration (MBC) of Cin, Cin-NE, and Cin-RHL-NE were determined using the microdilution and plate colony counting methods, in accordance with the standards of the American Society for Clinical Laboratory Standards (CLSI) (Arendrup et al., 2017).

##### 2.4.1.2 Electrical conductivity assay

The USA300 bacterial suspension (OD_600_ = 1.8), cultured to the logarithmic phase, was inoculated with 2% inoculum in TSB medium and incubated in a gas-bath thermostatic shaker at 37°C for 16 h. After incubation, the USA300 cultures were divided into five groups and subjected to various treatments: Cin (1,024 μg/mL), Cin-NE (Cin, 1,024 μg/mL), Cin-RHL-NE (Cin, 1,024 μg/mL), Blank-RHL-NE, and PBS. The conductivity of the different treatment groups was measured using a conductivity meter (NanoDrop One, Thermo Scientific, Waltham, MA, USA) (Hassan et al., 2020). Each treatment was performed in triplicate.

##### 2.4.1.3 Determination of MRSA DNA extravasation

USA300 bacterial suspension (OD_600_ = 1.8) was cultured to the logarithmic phase. The bacterial solution was then washed twice with PBS and re-suspended in PBS to a concentration of 10^7^ CFU/mL. Cin, Cin-NE, and Cin-RHL-NE were added to the treatment groups at a final concentration of 1,024 μg/mL. The treatment groups were incubated at 37°C for 0, 1, 2, 4, 6, and 8 h. After incubation, the samples were centrifuged at 4500 rpm for 10 min, and the supernatants were collected. DNA concentrations in the supernatants were determined using a microspectrophotometer at OD_260nm_ (NanoDrop One, Thermo Scientific, Waltham, MA, USA) (Lin et al., 2018).

##### 2.4.1.4 Effect of NEs on membrane permeability and fluidity of MRSA cells

USA300 bacterial suspension (OD_600_ = 1.8) was centrifuged at 4500 rpm for 10 min. Following centrifugation, the PI (Solarbio, Beijing, China) probe was added to the USA300 bacteria. The fluorescence intensity of the bacteria was then measured using a microplate reader (Thermo Scientific, Waltham, MA, USA) with an excitation wavelength of 488 nm and an emission wavelength of 617 nm (Ye et al., 2023).

USA300 bacterial suspension (OD_600_ = 1.8) was centrifuged at 4500 rpm for 10 min. The working solution of the Luardan probe (Solarbio, Beijing, China) was then mixed with the USA300 bacterial solution, which had a bacterial cell concentration of approximately 1 × 10^6^ CFU/mL, according to the method described by Dubinin et al. (2020). The fluorescence intensities were measured using a microplate reader (Thermo Scientific, Waltham, MA, USA) with excitation and emission wavelengths set at 360 and 470 nm, respectively. GP values were calculated following the method proposed by Scheinpflug et al. (2017).

##### 2.4.1.5 Effects of NEs on the morphology of MRSA cell membranes

The bacteria (1 × 10^6^ CFU/mL) were treated with 2.5% glutaraldehyde fixative (Solarbio, Beijing, China) for 12 h after Cin, Cin-NE, Cin-RHL-NE was added to treatment groups at a final concentration of 1,024 μg/mL, dehydrated in graded ethanol, dried at critical point, sputter-coated with gold, and then observed by scanning electron microscopy (SEM) (Hitachi S4800N; Tokyo, Japan) (Inoue et al., 2017).

##### 2.4.1.6 Examination of MRSA live/dead ratios

The bacteria were stained with SYTO 9 and PI dyes (Maokang, Shanghai, China) for 30 and 15 min, respectively, and visualized by confocal laser scanning microscope (CLSM) (Sp8,Leica, Germany) (Su et al., 2023).

#### 2.4.2 Determination of NEs’ anti MRSA biofilm activity

#### 2.4.3 Biofilm quantification

Overnight cultured MRSA and 3% sucrose BHI medium (1:10) were added to 24-well plates and incubated at 37°C for 24 h to form MRSA biofilms. Cin-NE and Cin-RHL-NE were added at concentrations of 0, 128, 256, 512, and 1,024 μg/mL. The samples were rinsed with PBS to remove unbound substances, fixated with formaldehyde, and subsequent stained with crystal violet to visualize the adherent biofilms. Thereafter, the stained biofilms were dissolved using 30% acetic acid, and the absorbance at a wavelength of 590 nm was measured using a microplate reader (Peng et al., 2020). After determining the optimal treatment concentration, different group treatments were performed (including Cin, Cin-NE, Cin-RHL-NE, Blank-RHL-NE and PBS; the concentration of Cin was 1,024 μg/mL). Crystal violet staining was repeated and the amount of biofilm residue was quantitatively determined post-treatment. Each group was set in triplicate.

##### 2.4.3.1 Determination of extracellular polysaccharide content in MRSA biofilms

Phenol-sulfuric acid method was used to establish the concentration versus absorbance curve of glucose at OD_490_ nm. The biofilm-culture method was the same as described previously. The phenol-sulfuric acid method was used to extract the extracellular polysaccharides from the biofilm after treatment of Cin-NE, Cin-RHL-NE (the concentration of Cin was 0, 128, 256, 512, 1024 μg/mL), and the absorbance at OD_490_ nm was measured by microplate reader. Repeat the above process with different treatment groups including Cin, Cin-NE, Cin-RHL-NE, Blank-RHL-NE, and PBS (the concentration of Cin was 1,024 μg/mL). Each group was set in triplicate.

##### 2.4.3.2 Determination of MRSA biofilms extracellular protein content

The relationship between protein concentration and absorbance was plotted using a BCA protein kit (Beyotime, Shanghai, China). A MRSA biofilms was cultured as previously described. The extracellular proteins of the biofilm were extracted by ultrasonication (40 Hz, 20 min) after treatment of Cin-NE, Cin-RHL-NE (the concentration of Cin was 0,128,256,512,1,024 μg/mL), and the extracellular protein content of each group was determined using a BCA protein kit. Repeat the above process with different treatment groups including Cin, Cin-NE, Cin-RHL-NE, Blank-RHL-NE, and PBS (the concentration of Cin was 1,024 μg/mL). Each group was set in triplicate.

##### 2.4.3.3 Examination of MRSA biofilm live/dead ratios

Cin (1,024 μg/mL), Cin-NE (Cin,1,024 μg/mL), Cin-RHL-NE (Cin,1,024 μg/mL), Blank-RHL-NE, and PBS were added to the treatment groups, respectively. Then each group were stained with SYTO 9 and PI dyes for 90 min and observed under CLSM (Li et al., 2023).

##### 2.4.3.4 eDNA levels assay of MRSA biofilm

Cin (1,024 μg/mL), Cin-NE (Cin,1,024 μg/mL), Cin-RHL-NE (Cin,1,024 μg/mL), RHL-Blank-NE, and PBS were added to the treatment groups, respectively. Then each group were stained with SYTOX (Maokang, Shanghai, China) for 30 min and observed under CLSM (Su et al., 2023).

##### 2.4.3.5 Examination of MRSA biofilm surface structures

Each group was treated for 12 h with 2.5% glutaraldehyde fixative, dehydrated in an ethanol gradient, dried at the critical point, sputter-coated with gold, and then observed with SEM (Gu et al., 2022).

### 2.5 Evaluation of anti-biofilm effects *in vivo*

#### 2.5.1 Animals and experimental design

All animal procedures were reviewed and approved by the Animal Care and Use Committee of Sichuan Agricultural University (2017-0608). SPF BALB/c mice (8-weeks old, 18–20 g) were purchased from Dossy Experimental Animals Co., Ltd., (Chengdu, China). The animals were provided with water and mouse chow, and were maintained in a specific pathogen-free environment, with a 12 h light–dark cycle and 22 ± 1°C room temperature. After acclimatization under free access to food and water for 7 days, the infection model was created by implanting biofilm carriers on the backs of mice (Ji et al., 2016). The biofilm was cultured on a plastic sheet in a 12-well plate for 24 h. The sheet was then rinsed with PBS to remove free bacteria and implanted subcutaneously into the back of anesthetized BALB/c mice. The skin was cut open, the plastic sheet with MRSA biofilm was implanted, and the skin was then sutured closed.

48 h after implantation, mice were randomly divided into 5 groups (6 mice each group) as follows. The Group I was model group (Model), the Group II was RHL-Blank-NE group, and the Group III-V were supplemented of 100 μLCin, Cin-NE and Cin-RHL-NE at a Cin concentration of 40 mg/kg. All regents were injected once daily subcutaneously for 7 consecutive days.

#### 2.5.2 Hematological and tissue testing

An aliquot of the blood of the mice from the experimental groups of the mice skin implantation healing model was collected to quantify the hematological indexes. The blood was transferred to tubes with anticoagulant and the leukocytes and neutrophils analyses were measured on the hematimeter (BC-2800Vet; Mindray, Shenzhen, China). TNF-*α* and IL-6 levels were also detected by ELISA kits (Lianke, Hangzhou, China).

Mice dorsal skin tissue was excised and subsequently homogenized for the enumeration of infected skin colonies (Tursi et al., 2020).

Skin tissue proximal to the infected lesion on the dorsum of mice was excised and stabilized in 4% paraformaldehyde (Solarbio, Beijing, China). Histological sections subjected to Hematoxylin and Eosin (H&E) staining were then examined to assess pathological changes, thereby elucidating the anti-inflammatory effectiveness of the administered treatments.

#### 2.5.3 CLSM analysis of eDNA in biofilms on implant surfaces

The implant was extracted from the mice, and the extracellular DNA (eDNA) present within the biofilm coating the implant surface was labeled with SYTOX (Maokang, Shanghai, China) for an hour. Subsequently, visualization and examination of the fluorescently stained eDNA were conducted using CLSM (Li et al., 2023).

### 2.6 Statistical analysis

The results are shown as the mean and standard deviation of the measured values. Statistical analysis was performed using Origin 2018, IBM SPSS Statistics, Graphpad Prism 10. To explore the differences between groups, we analyzed the data by ANOVA, and *p* < 0.05 was considered statistically significant.

## 3 Results

### 3.1 Screening and optimization of prescription

#### 3.1.1 Screening of oil phase, emulsifiers and co-emulsifiers and Km

**Figure 1:**
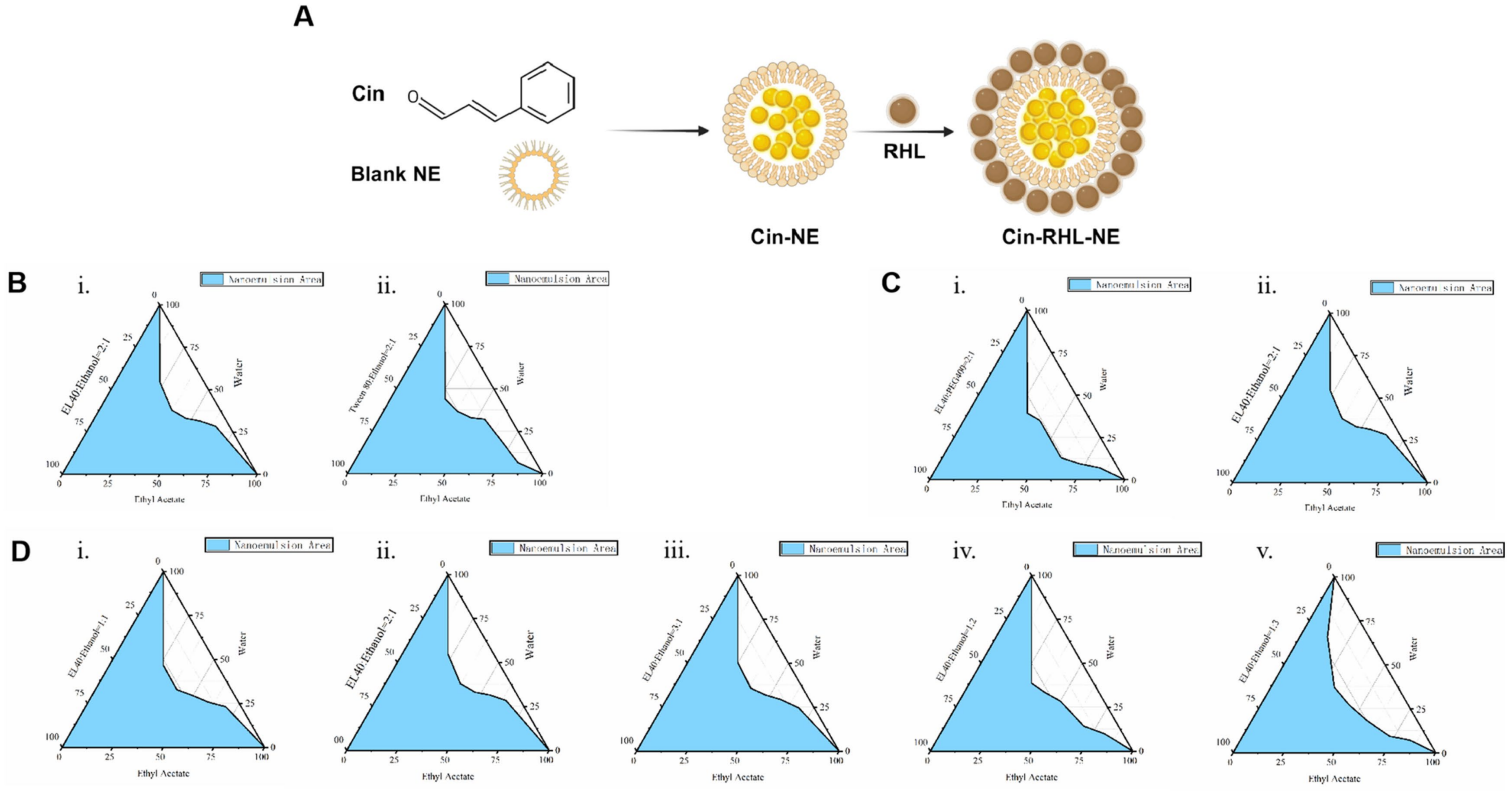
Preparation of Cin-NE. **(A)** Diagram of Cin-RHL-NE. **(B)** Ternary phase diagrams of screening of emulsifiers. **(C)** Ternary phase diagrams of screening of co-emulsifiers. **(D)** Ternary phase diagrams of screening of Km.

The NEs was prepared as the method in 2.2.1. The Schematic diagram of Cin-RHL-NE was shown in Figure 1A. The results showed that the mixture of cinnamaldehyde and ethyl acetate was stable in state and did not stratify. Therefore ethyl acetate is used as the oil phase in nanoemulsion systems due to its good solubility and efficient dispersibility. Emulsifiers and co-emulsifiers are pivotal components in the nanoemulsion fabrication process, functioning by adsorbing at fluid interfaces to diminish interfacial tension and facilitate the formation of stable particles.

The larger the formed nanoemulsion zone, the better the emulsifying ability. By comparing the nanoemulsion zones (the zone colored blue in Figure 1B) formed by EL-40 (Figure 1Bi) and Tween 80 (Figure 1Bii), it is observed that the nanoemulsion zone formed by EL-40 is larger. Therefore, EL-40 is selected as the emulsifier for this nanoemulsion.

The principle of selecting an co-emulsifier is consistent. Ethanol, when used as a co-emulsifier, forms a larger area of nanoemulsion region compared to PEG 400 used as a co-emulsifier (Figure 1C). Therefore, ethanol is chosen as the co-emulsifier for this nanoemulsion composition.

Moreover, the ternary phase diagram (Figure 1D) illustrated that the most extensive emulsion region is attained when the Km ratio, representing the balance between emulsifier and co-emulsifier, is set at 2:1. Consequently, a Km value of 2:1 was adopted to maximize the stability and performance of nanoemulsion.

#### 3.1.2 Optimization of nanoemulsion prescription

**Figure 2:**
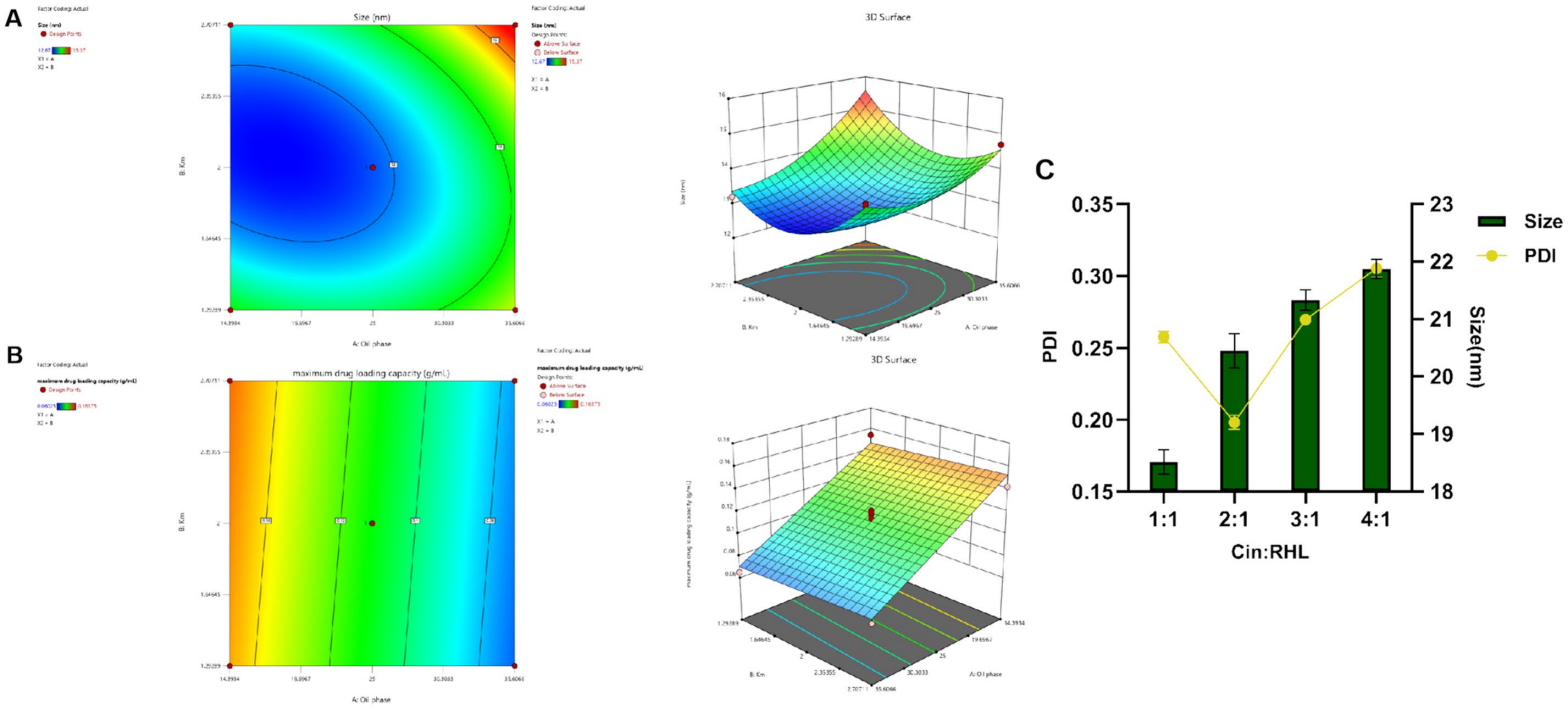
Optimization of nanoemulsion prescription. **(A,B)** Response surface and contour plots of the interactions between oil phase volume and Km value on the particle size and maximum encapsulation capacity of the Blank-NE. **(C)** Screening of RHL dosage based on particles size and PDI.

CCD-RSM was utilized to investigate the influence of the oil phase (X1) and Km value (X2) on particle size (Y1) and maximum drug-loading capacity (Y2). The Design Expert 13.0 software was employed for experimental design and analysis using the CCD-RSM (Figures 2A,B). The factors, levels, experimental arrangements, and results are presented in Table 2.

**Table 2:** CCD-RSM factors, levels, and outcomes.

Group	X1:Oil phase	X2:Km	Y1:Size/nm	Y2:maximum drug-loading capacity/g/mL
1	25.00	2.00	12.92	0.1178
2	25.00	2.00	13.01	0.1215
3	25.00	3.00	15.2	0.1153
4	25.00	1.00	14.45	0.0953
5	10.00	2.00	12.98	0.1638
6	35.61	1.29	14.71	0.0650
7	25.00	2.00	12.99	0.1198
8	14.39	2.71	13.21	0.1423
9	25.00	2.00	12.86	0.1083
10	14.39	1.29	14.2	0.1543
11	40.00	2.00	14.97	0.0603
12	25.00	2.00	12.67	0.1143
13	35.61	2.71	15.37	0.0728

#### 3.1.3 Model fitting

The model was fitted post experimental results, with the regression equation obtained through Design Expert 13.0 software. The particle size model fit *F* = 52.94, *p* < 0.0001 < 0.05, indicating well fitted and predictive. The maximum drug-loading capacity complied with the multivariate linear equation. The nonlinear fit of particle size was better, with correlation coefficients high and results statistically significant. From the 3D plots and equations, the final values of factors X1 and X2 were derived using the prediction function of Design-Expert 13.0: X1 = 14.393% and X2 = 2.151, and the result X2 = 2.151 is similar to the previous Km value screening result, which is in line with the expectation. Ethyl acetate accounted for 14.393%, EL-40 58.439%, and ethanol 27.168%.

#### 3.1.4 Optimal prescription validation

The validation results showed a small deviation between the true and predicted values, indicating CCD had good predictive effect.

#### 3.1.5 Dosage of Cin and RHL

Preliminary validation experiments indicated that the upper limit for Cin incorporation was 0.15675 g/mL. However, subsequent trials revealed a propensity for nanoemulsion instability when the Cin concentration exceeded 0.05 g/mL. Consequently, based on these observations, the optimal and practical amount of Cin to ensure nanoemulsion stability was established at 0.05 g/mL.

Assessment of RHL dosage revealed that an increase in the proportion of Cin led to elevated particle sizes yet concurrently changed PDI values (Figure 2C). As the particle size decreases, the smaller-sized droplets having greater surface area providing greater absorption. A smaller PDI indicated a more uniform relative particle mass distribution and higher particle size uniformity (Donsi et al., 2012). Cinnamaldehyde, serving as the core of the nanoemulsion, increased in particle size with greater amounts. Additionally, rhamnolipid, used as a modifier, if present in insufficient quantities, fails to decorate the particle surfaces adequately, leading to an increase in PDI and uneven particle sizes. Considering the pivotal roles of nanoemulsion stability and particle size uniformity, the optimum Cin: RHL ratio was established to be 2:1, thereby striking a balance between effective incorporation and maintaining the desired physicochemical properties.

Based on the above results, ethyl acetate was selected as the oil phase of the Cin-NE, EL40 as the emulsifier of the Cin-NE, and ethanol as the coemulsifier of the Cin-NE with Km of 2.151. Each 3 g of Cin-NE contained 0.15 g Cin, 0.28 g ethyl acetate, 1.174 g EL40, 0.546 g ethanol, and 0.85 g distilled water. CIN-RHL-NE adds 0.075 g RHL to Cin-NE.

### 3.2 Characterization of Cin-NE and Cin-RHL-NE

#### 3.2.1 Emulsion type identification, particle size, zeta potential and morphology

**Figure 3:**
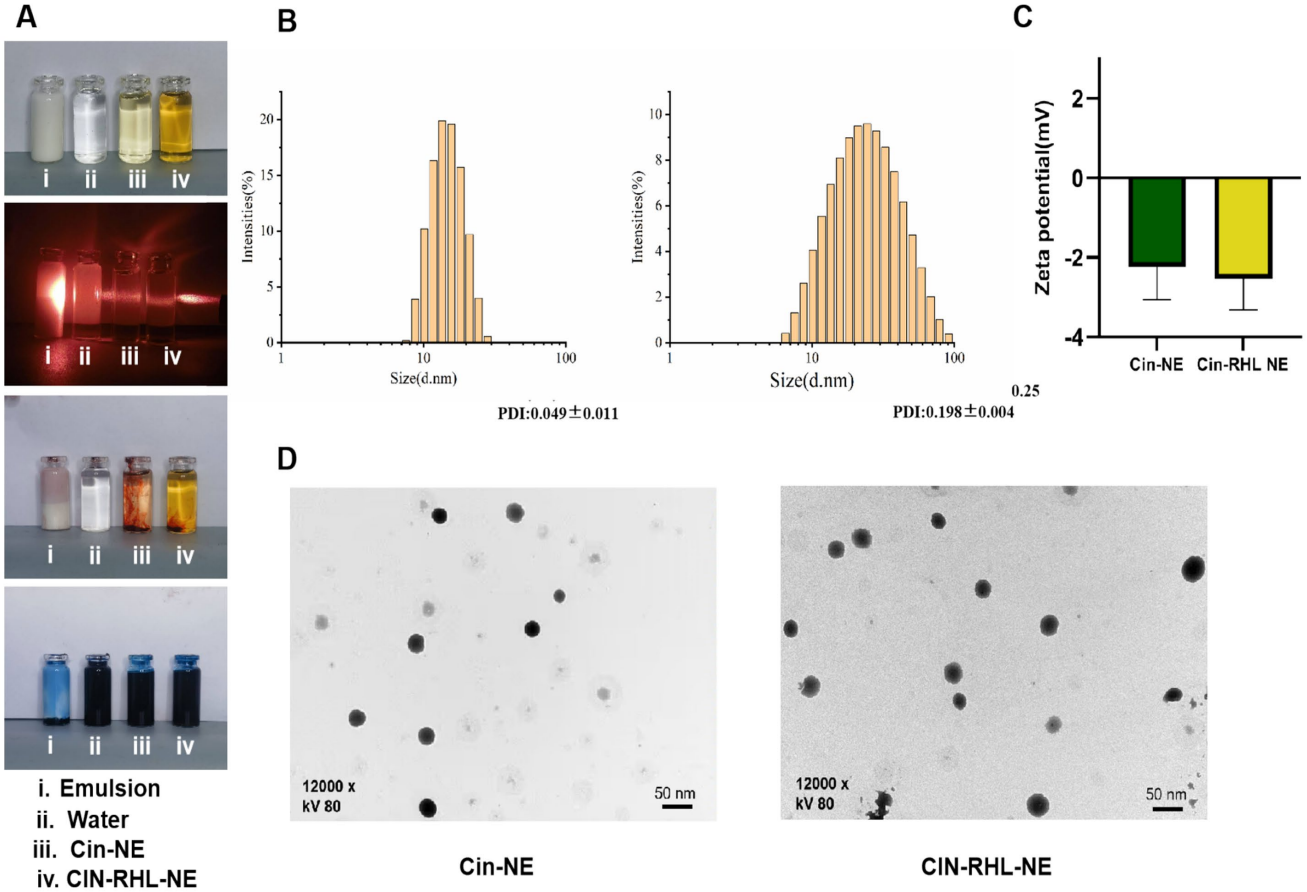
Characterization of nanoemulsions. **(A)** Physical appearance of the NEs; Visualization of the Tyndall effect; Type identification using Sudan III and Methylene Blue dyes. **(B)** Particle size distribution of Cin-NE and Cin-RHL-NE. **(C)** Zeta potentials of Cin-NE and Cin-RHL-NE. **(D)** TEM morphologies of Cin-NE and Cin-RHL-NE particles.

Under (25 ± 1)°C, the Cin-NE was a transparent, homogeneous, and stable yellow liquid system, while the Cin-RHL-NE was a light brown, uniformly transparent liquid system. Under laser irradiation, both NEs exhibited Tyndall effect, indicating particles sizes in the range of 1–100 nm. Additionally, the observation that the water-soluble dye (methylene blue) diffused more rapidly in comparison to the oil-soluble dye (Sudan III) in both nanoemulsion systems, strongly suggested their composition as oil-in-water (O/W) type NEs (Figure 3A).

The size of Cin-NE was 13.66 ± 0.08 nm (Figure 3B1) and the size of Cin-RHL-NE was 20.45 ± 0.25 nm (Figure 3B2), with the PDI of Cin-NE was 0.049 ± 0.011 and the PDI of Cin-RHL-NE was 0.198 ± 0.004, respectively. The results indicated that the novel formulation of NEs achieved a small particle size. The zeta potential of Cin-NE was −2.24 ± 0.67 mV and the zeta potential of Cin-RHL-NE was −2.53 ± 0.4 mV (Figure 3C).

TEM showed (Figure 3D) that the particle morphology of both NEs exhibited spherical shapes featuring smooth edges and a high degree of size uniformity.

#### 3.2.2 Encapsulation rate and drug loading capacity

The standard curve and dilution multiplicity relationship were used to measure the absorbance of the corresponding solution by UV spectrophotometer. From this, the total active substance (Wt) and the concentration of the free active ingredient (W_f_) were calculated. The encapsulation efficiency of Cin-NE was (99.84 ± 0.09)%.

#### 3.2.3 Stabilities of nanoemulsions

**Figure 4:**
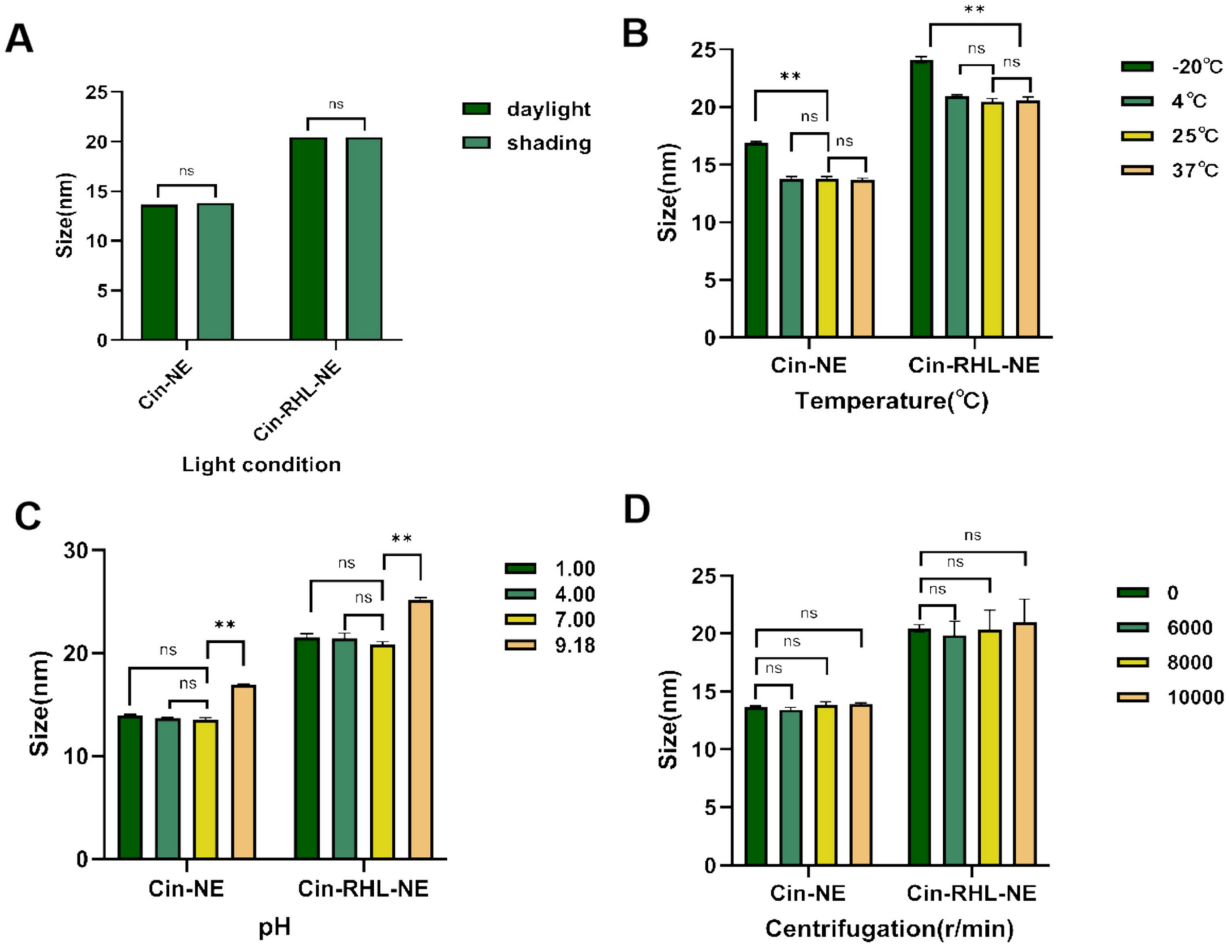
Stabilities of nanoemulsions. **(A)** Effect of light conditions (illuminated at 400Lx and dark storage conditions at 0Lx) on the mean particle diameter of Cin-NE and Cin-RHL-NE. ns denoted no significant difference between light and dark storage conditions (*p* > 0.05). **(B)** Influence of storage temperatures (−20°C, 4°C, 25°C, 37°C) on the mean particle diameter of Cin-NE and Cin-RHL-NE. ns denoted no significant difference compared to 25°C (*p* > 0.05),** denoted a significant difference compared to 25°C (*p* < 0.01). **(C)** Effect of pH levels on the mean particle diameter of Cin-NE and Cin-RHL-NE. ns denoted no significant difference compared to pH 7 (*p* > 0.05), and ** denoted a significant difference relative to pH 7 (*p* < 0.01). **(D)** Influence of centrifugation on the mean particle diameter of Cin-NE and Cin-RHL-NE. ns denoted no significant difference compared to non-centrifugation (*p* > 0.05).

Figures 4A,B showed that there were no statistically significant differences within the 30-days period of storage at 4°C, 25°C, and 37°C under both shading (0 Lx) and daylight (400 Lx). The increase in particle size at −20°C, significantly different from 25°C (*p* < 0.05), suggested NEs were not stable for preservation at −20°C while indoor temperature and simulated body temperature conditions were stable for preservation.

The results of centrifugation tests (Figure 4D) demonstrated that neither the particle size nor PDI of Cin-NE and Cin-RHL-NE were significantly affected when subjected to centrifugation speeds of 6,000 r/min, 8,000 r/min and 10,000 r/min (*p* > 0.05).

Results showed that there were no significant differences in particle size of the NEs under both acidic and neutral conditions (Figure 4C) indicating they could be stabilized in these environments.

### 3.3 *In vitro* antimicrobial activity

#### 3.3.1 NEs performed better on the inhibiting growth of USA300 than Cin

The MIC values of Cin-RHL-NE and Cin-NE on MRSA were both measured as 512 μg/mL and MBC values were both 1,024 μg/mL according to the micro-broth dilution method in the CLSI standard; whereas, the MIC values of Cin on MRSA were 1,024 μg/mL and MBC values were 2048 μg/mL.

#### 3.3.2 Planktonic bacteria

##### 3.3.2.1 The USA300 extracellular DNA release and conductivity of NEs group was increased

**Figure 5:**
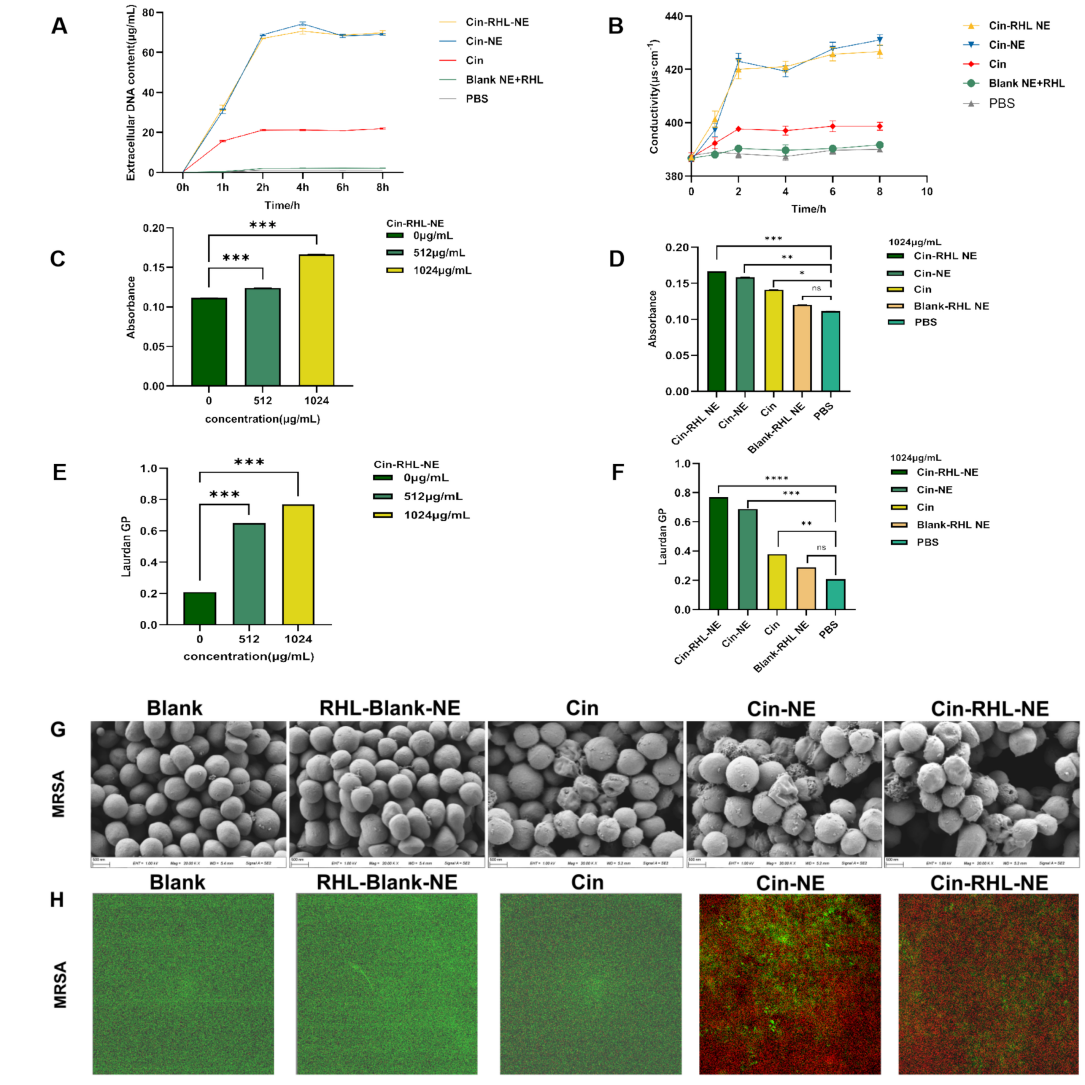
*In vitro* antimicrobial activity of NEs on planktonic USA300. **(A)** Impact of different treatment groups on the extracellular DNA release of USA300. **(B)** Effect of various treatment groups on the conductivity of USA300 suspensions. **(C)** Influence of different concentrations of Cin-RHL-NE on the permeability of MRSA cell membranes. **(D)** Effect of diverse treatment regimens on the permeability of MRSA cell membranes. **(E)** Concentration-dependent influence of Cin-RHL-NE on the fluidity of MRSA cell membranes. **(F)** Impact of different treatment groups on the fluidity of MRSA cell membranes. **(G)** SEM images of MRSA post various treatments. Scale bar, 500 nm. **(H)** CLSM images of MRSA following different treatments, with green fluorescence indicating live bacteria stained with Syto9 and red fluorescence representing dead bacteria stained with PI. *p < 0.05, **p < 0.01, ***p < 0.001, using Student’s t-test.

From Figure 5A, it was evident that following a 2-h treatment of the regnents to USA300 DNA, the release of DNA from the cells increased significantly. Specifically, the Cin-RHL-NE group, Cin-NE group, and Cin group exhibited respective enhancements of 67.773 ± 0.295 μg/mL (*p* < 0.001), 65.971 ± 0.107 μg/mL (*p* < 0.001), and 20.109 ± 0.241 μg/mL (*p* < 0.001) in comparison to the control group. Notably, both the Cin-RHL-NE and Cin-NE formulations demonstrated superior efficacy over the Cin group alone.

As could be seen from the Figure 5B, the conductivity values of Cin-RHL-NE, Cin-NE, and Cin group after 6 h treatment was 425.67 ± 2.05 μS·cm^−1^, 427.67 ± 2.05 μS·cm^−1^, and 398.67 ± 1.70 μS·cm^−1^, respectively. Compared to the control group’s baseline conductivity of 389.67 ± 1.25 μS·cm^−1^, these readings marked increased of 9.23% (*p* < 0.01), 10.25% (*p* < 0.01), and 2.31%, respectively. It showed that the conductivity of USA300 bacterial suspension was significantly increased after treatment with Cin-RHL-NE and Cin-NE. Both Cin-RHL-NE and Cin-NE formulations have effectively elevated the membrane permeability of MRSA cells.

##### 3.3.2.2 Qualitative analysis of USA300 cell membrane permeability and cell membrane fluidity

The fluorescent dye PI could enter the bacterium through the cell membrane, and its fluorescence within bacteria can be quantified at specific excitation and emission wavelengths. Leveraging this, the fluorescence intensity of PI was employed to probe the impact of varying concentrations of Cin-RHL-NE (512 μg/mL-1024 μg/mL) and different treatment regimens on the permeability of USA300 cell membranes. As illustrated in the (Figure 5C), an ascending trend in PI fluorescence intensity paralleled the increase in Cin-RHL-NE concentration, suggesting a concentration-dependent effect of Cin-RHL-NE on MRSA membrane permeabilization. In a separate assessment, MRSA was treated with different formulations at a fixed Cin concentration of 1,024 μg/mL. The outcomes revealed no statistically significant variation between the Blank-RHL-NE group and the control group (*p* > 0.05). Conversely, the fluorescence intensities for the Cin-RHL-NE, Cin-NE, and Cin groups were augmented by 0.055, 0.047, and 0.003 relative to the control group, respectively, manifesting statistically significant differences (*p* < 0.05). Notably, the increments for both the Cin-RHL-NE and Cin-NE groups were found to be significantly higher (*p* < 0.001) than that of the Cin group alone, underscoring the superior performance of the nanoemulsion formulations in enhancing MRSA membrane permeability (Figure 5D).

The GP value of Luardan, a hydrophobic fluorescent dye, indicated the order or disorder of nearby phospholipids in cell membranes. An increased GP value implies enhanced membrane order and rigidity, and hence diminished fluidity. The effect of Cin-RHL-NE on the fluidity of MRSA cell membranes was concentration-dependent, with a Laurdan GP of 0.77 ± 0.01 at a concentration of 1,024 μg/mL, compared with 0.21 ± 0.01 in the control group, which significantly reduced the fluidity of the cell membranes (Figure 5E). Cin-RHL-NE exerted a pronounced concentration-dependent impact on the fluidity of USA300 cellular membranes, causing a significant decrease at a 1,024 μg/mL concentration (Figure 5F). In contrast, Cin and the Blank-RHL-NE did not yield significant alterations. These findings affirmatively highlighted the specific effect of Cin-RHL-NE on modulating USA300 membrane fluidity, outperforming Cin alone in its potency.

##### 3.3.2.3 SEM observation of USA300 morphology

SEM revealed a smooth, regular surface in the control group, with few wrinkles. Conversely, the USA300 in the Cin group showed some wrinkles and irregular morphology. The bacterial morphology of the Cin-RHL-NE and Cin-NE groups was severely affected, manifesting wrinkles, collapses and ruptures. The contents of the bacteria flowed out, indicating serious damage to MRSA. There was no significant difference in the Blank-RHL-NE group compared to the control group (Figure 5G).

##### 3.3.2.4 CLSM observation of NEs-killed USA300

The results from the CLSM images (Figure 5H) showed that the control group exhibited mostly green fluorescence, indicating a high percentage of live bacteria. The Blank-RHL-NE group also showed largely green fluorescence, suggesting no killing effect on MRSA. The Cin group had some red fluorescence, indicating a limited killing effect on MRSA at this concentration. The CLSM results of Cin-RHL-NE and Cin-NE groups showed almost entirely red fluorescence, proving both could kill nearly all MRSA.

#### 3.3.3 Antibiofilm activity

##### 3.3.3.1 Inhibition of biofilm formation

**Figure 6:**
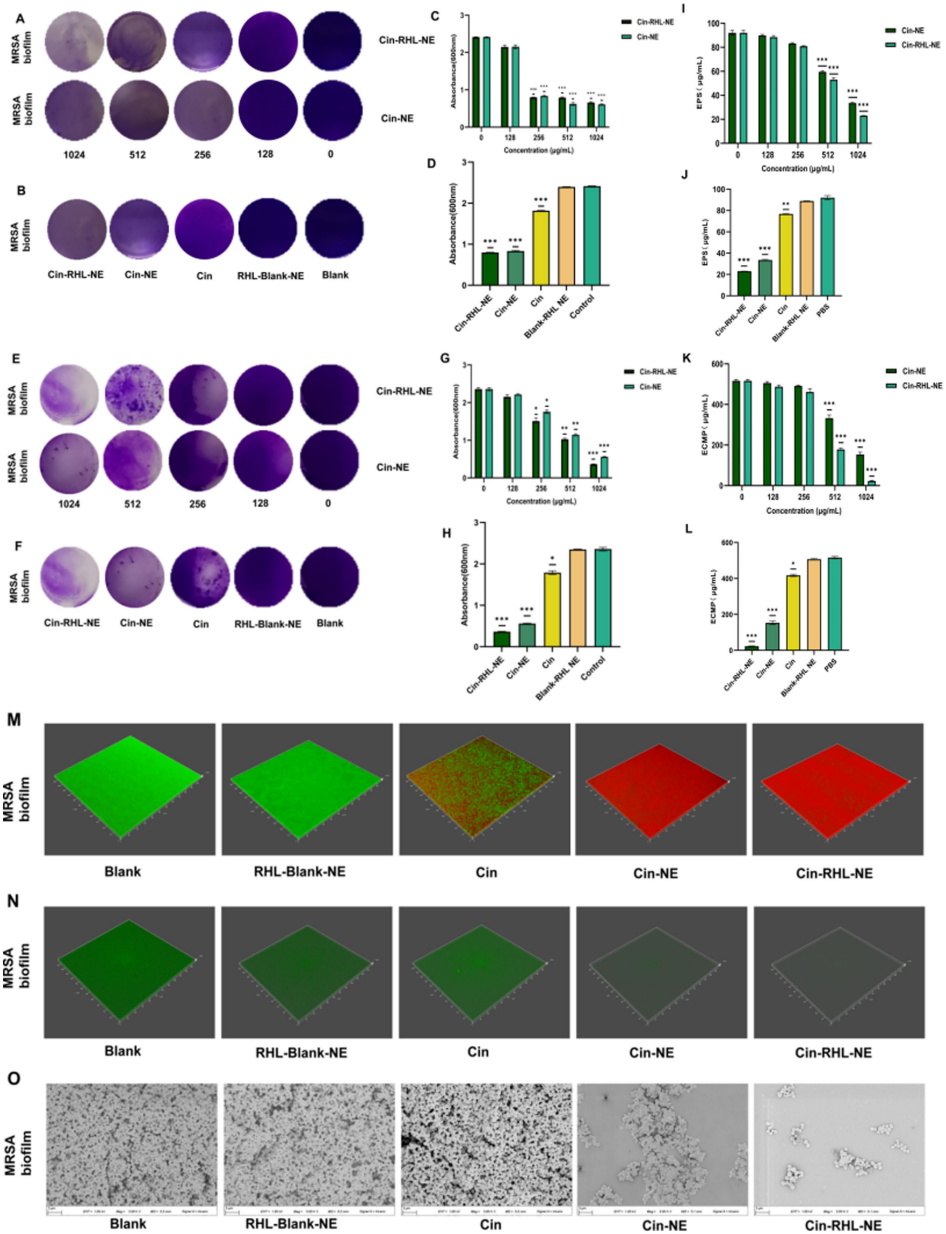
*In vitro* assessment of MRSA biofilm eradication. **(A)** Crystal violet-stained images of MRSA biofilms after treatment with varying concentrations of Cin-RHL-NE and Cin-NE. **(B)** Crystal violet-stained images of MRSA biofilms post different treatments. **(C)** Biomass of MRSA biofilms following treatment with differing concentrations of Cin-RHL-NE and Cin-NE. **(D)** Biomass measurements of MRSA biofilms after various treatments. **(E)** Crystal violet-stained images showcasing the impact of Cin-RHL-NE and Cin-NE at varied concentrations on MRSA biofilms. **(F)** Eradication images of crystal violet-stained MRSA biofilms post different treatments. **(G)** Quantification of biomass from MRSA biofilms post-treatment with different concentrations of Cin-RHL-NE and Cin-NE. **(H)** Biomass assessment of MRSA biofilms after various therapeutic interventions. **(I)** Effects of different concentrations of Cin-RHL-NE and Cin-NE on the extracellular polysaccharide content of MRSA biofilms. **(J)** Analysis of the impact of various treatments on the extracellular polysaccharide content in MRSA biofilms. **(K)** Examination of the influence of variable concentrations of Cin-RHL-NE and Cin-NE on the extracellular protein content of MRSA biofilms. **(L)** Assessment of the effect of different treatments on the extracellular protein content in MRSA biofilms. **(M)** CLSM 3D reconstructions of MRSA biofilms post-treatment, where green fluorescence denotes live bacteria stained with SYTO 9 and red fluorescence represents dead bacteria stained with PI. **(N)** Detection of eDNA levels in MRSA biofilms using SYTOX staining. **(O)** Scanning Electron Microscopy (SEM) images of MRSA biofilms after various treatments, Scale bar, 3 μm. *p < 0.05, **p < 0.01, ***p < 0.001, using Student’s t-test.

Crystal violet staining demonstrated that both Cin-RHL-NE and Cin-NE inhibited MRSA biofilm formation in a concentration-dependent manner, with effective concentrations ranging from 128 to 1,024 μg/mL (Figure 6A). Specifically, Cin-RHL-NE reduced MRSA biofilm proliferation by 66.9% at a concentration of 256 μg/mL (Figures 6B,C). Based on this effective concentration, further investigations were conducted to evaluate the inhibition of various treatments on USA300 biofilm growth. The results indicated that both Cin-RHL-NE and Cin-NE significantly inhibited USA300 biofilm growth, with reductions of 66.9 and 65.5%, respectively. Both treatments showed statistically significant superiority over the control group. In contrast, Cin alone resulted in a much lesser inhibition, with only a 24.8% reduction in growth. The Blank-RHL-NE displayed negligible inhibitory efficacy (Figure 6D).

##### 3.3.3.2 Eradication of formed biofilm

Crystal violet staining showed that the scavenging effect of Cin-RHL-NE and Cin-NE on mature biofilm of MRSA showed concentration dependence at concentrations of 128–1,024 μg/mL (Figures 6E,G). The study demonstrated nanoemulsion effectively removed MRSA biofilm in a concentration-dependent manner. Specifically, Cin-RHL-NE exhibited the highest removal rate of 84.6% at 1024 μg/mL, while Cin-NE removed 76.3%. In contrast, Cin demonstrated significantly reduced efficiency, managing to eliminate merely 24.1% of the biofilm. The Blank-RHL-NE, devoid of the active ingredient, had a negligible impact on biofilm clearance (Figures 6F,H).

##### 3.3.3.3 Quantitative analysis analysis of extracellular polysaccharides and extracellular proteins of USA300 biofilms

The absorbance of glucose standard solutions was measured using UV spectrophotometry at OD_490_ nm, with the standard curve equation being *y* = 0.0276 x + 0.1766 (*R*^2^ = 0.9996). The impact of Cin-NE and Cin-RHL-NE on the extracellular polysaccharide content of USA300 was assessed using the phenol-sulfuric acid method. Results showed that as the drug concentration increased (0, 128, 256, 512, and 1,024 μg/mL), the extracellular polysaccharide content decreased (Figure 6I). At a Cin concentration of 1,024 μg/mL, the effects on the extracellular polysaccharide content of the USA300 biofilm were compared among Cin-RHL-NE, Cin-NE, Cin, Blank-RHL-NE, and PBS control. Compared to the control, the extracellular polysaccharide content was reduced by 69.07 ± 1.49 μg/mL (*p* < 0.001) and 58.54 ± 1.05 μg/mL (*p* < 0.001) for Cin-RHL-NE and Cin-NE, respectively, indicating that the NEs significantly reduced the extracellular polysaccharide content of USA300. The Cin group also demonstrated some reduction, but it was less effective compared to Cin-NE and Cin-RHL-NE. Blank-RHL-NE had minimal impact on MRSA’s extracellular polysaccharide content (Figure 6J).

The standard curve equation obtained with BCA protein kit was *y* = 0.0007 x + 0.1135 (*R*^2^ = 0.999). The BCA protein content was used to determine the extracellular protein content of USA300 treated with different concentrations of Cin-RHL-NE and Cin-NE. The results showed that the extracellular protein content decreased with increasing drug concentration (Figure 6K). The effect of different treatments on the change of extracellular protein content of USA300 biofilm was determined when the concentration of Cin was 1,024 μg/mL. Compared with the control group, the extracellular protein content of Cin-RHL-NE and Cin-NE decreased by 493.81 ± 3.56 μg/mL (*p* < 0.01) and 363.33 ± 4.02 μg/mL (*p* < 0.01), respectively (Figure 6L). Cin-RHL-NE had a better effect on the extracellular protein content of MRSA than Cin-NE. Cin had some effect on USA300 extracellular protein content while Blank-RHL-NE had no significant effect.

##### 3.3.3.4 CLSM observation of the NEs effect on USA300 biofilms

CLSM results revealed that the biofilm in the control group predominantly exhibited green fluorescence, indicating a high proportion of viable bacteria. In contrast, the Cin group displayed both red and green fluorescence, suggesting that while Cin was effective in eliminating some of the living bacteria within the biofilm, it did not eradicate all viable bacteria. Similarly, the Cin-NE group also exhibited a mix of red and green fluorescence, which indicated incomplete eradication of bacteria attached to the biofilm membrane. In contrast, the Cin-RHL-NE group primarily showed red fluorescence, reflecting a highly effective elimination of live bacteria from the biofilm structure (Figure 6M).

##### 3.3.3.5 CLSM observation of the NEs effect on USA300 biofilms

From the SYTOX-stained fluorescence images (Figure 6N), it is evident that neither Cin nor Blank-RHL-NE causes effective damage to eDNA. In contrast, Cin-RHL-NE and Cin-NE significantly reduce eDNA levels in MRSA biofilms. Further evidence supports the scavenging effects of Cin-RHL-NE and Cin-NE on MRSA biofilms.

##### 3.3.3.6 SEM observation of the NEs effect on USA300 biofilms

SEM results showed that the biofilm structure was dense in the control group. Treatment with Blank-RHL-NE caused no noticeable changes in biofilm structure. A significant decline in biofilm density was observed in the Cin group. Remarkably, both nanoemulsion-treated groups exhibited substantial disintegration of the membrane structure, indicating their disruptive effects on the biofilm. The Cin-RHL-NE group demonstrated superior outcomes compared to Cin-NE, suggesting a heightened efficacy in degrading the biofilms (Figure 6O).

### 3.4 Evaluation of *in vivo* antibacterial activity

#### 3.4.1 Hematological and tissue test of the NEs treatment in mice implantation healing model

**Figure 7:**
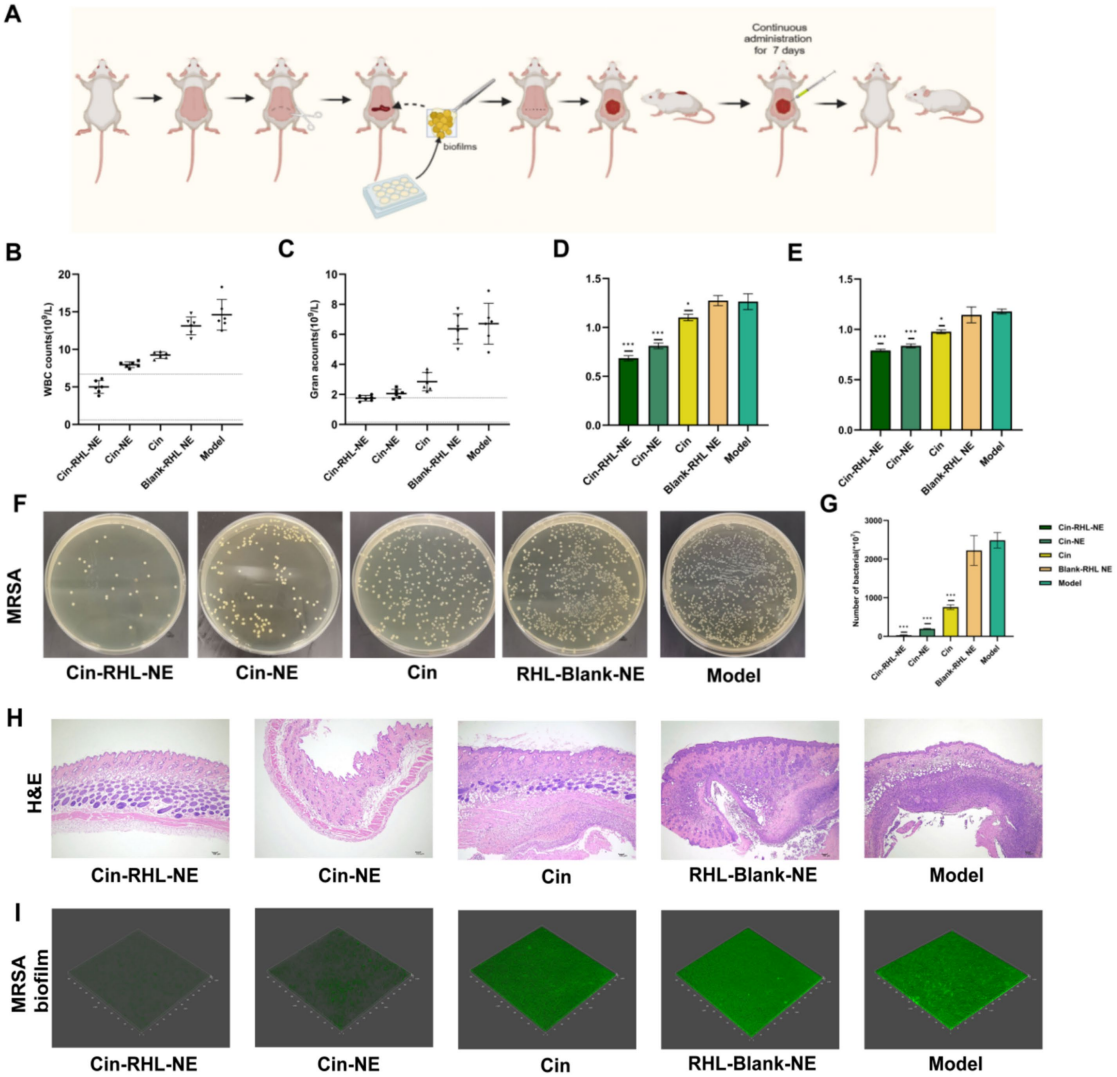
*In vivo* assessment of MRSA biofilm clearance in a mouse dorsal implant infection model. **(A)** Schematic illustrating the implantation of MRSA biofilm onto the mouse back and subsequent treatment regimen. **(B,C)** White blood cell and neutrophil counts in mice. **(D,E)** Serum levels of TNF-*α* and IL-6 in mice following different treatments. **(F)** Representative images of bacterial smears from excised mouse skin tissue. **(G)** Bacterial colony counts from mouse skin tissue. **(H)** Histopathological analysis of infected skin tissues via H&E staining, revealing morphological changes and inflammation status; Scale bar, 100 μm. *p < 0.05, **p < 0.01, ***p < 0.001, using Student’s t-test. **(I)** Detection of extracellular DNA (eDNA) levels in MRSA biofilm on implantation using SYTOX staining.

The construction of the mouse model and the treatment schematic are shown in Figure 7A. Leukocyte count results indicated a decrease in the Cin, Cin-NE, and Cin-RHL-NE treatment groups compared to the model group. Notably, the leukocyte count in the Cin-RHL-NE treatment group was restored to the physiologically normal range (reference value: 0.8–6.9 × 10^9/L). Similarly, the neutrophil count followed a comparable trend to the leukocyte count results (Figures 7B,C).

The results showed that, compared to the model group, the levels of TNF-*α* (Figure 7D) and IL-6 (Figure 7E) decreased in the Cin, Cin-NE, and Cin-RHL-NE treatment groups. Notably, the Cin-RHL-NE group experienced a significant reduction of 85.3% (*p* < 0.01) in TNF-α and 47.1% (*p* < 0.01) in IL-6, highlighting its superior healing impact.

Bacterial counts in the Cin, Cin-NE, and Cin-RHL-NE groups decreased significantly compared to the model group. Specifically, the Cin-NE group and Cin-RHL-NE group saw reductions of up to 12-fold and 87-fold, respectively, demonstrating the nanoemulsions’ effective *in vivo* antimicrobial properties (Figures 7F,G).

Infected skin tissues were collected and H&E stained to analyze morphological changes and the degree of inflammation. The model group exhibited severe infection symptoms, characterized by extensive infiltration of inflammatory cells into the subcutaneous tissues. In contrast, the Cin group showed reduced inflammation, though it remained noticeable. The Cin-NE treatment groups significantly improved infection characteristics and decreased the presence of inflammatory cells. Notably, the Cin-RHL-NE group achieved near-complete resolution of inflammation, with only a few residual inflammatory cells. Overall, the nanoemulsion preparation effectively addressed MRSA biofilm infection-induced (Figure 7H).

#### 3.4.2 Detection of eDNA in biofilms on implant surfaces

The SYTOX dye specifically binds to biofilm eDNA. CLSM results (Figure 7I) revealed strong green fluorescence in the model group, indicating minimal biofilm clearance. In contrast, fluorescence intensity decreased in the Cin, Cin-RHL-NE, and Cin-NE groups, with the fluorescence being nearly undetectable in the Cin-RHL-NE group. This observation confirms effective biofilm removal *in vivo*.

## 4 Discussion

Treating methicillin-resistant *Staphylococcus aureus* (MRSA), which is notorious for its treatment-resistant biofilms, presents significant challenges. This underscores the need for innovative, compound-focused therapeutic approaches to effectively eradicate these biofilms. Recent research has highlighted the potential of various natural compounds, such as limonene, andrographolide sulfonate, and luteolin, in impeding biofilm formation (Gambino et al., 2022; Yuan et al., 2022; Zhang et al., 2021). Given cinnamaldehyde (Cin) is established as a broad-spectrum antimicrobial agent with potent inhibitory capabilities (Shen et al., 2015), our study aimed to explore its effectiveness when formulated into a nanoemulsion. We also investigated the potential of combining Cin with rhamnolipid (RHL) to specifically target and disrupt biofilms.

Our investigation revealed notably promising antibacterial activities from the fabricated nanoemulsions, with Cin-NE demonstrating the highest inhibitory efficacy. The potency hierarchy observed was led by Cin-NE, which had a minimal inhibitory concentration (MIC) of 512 μg/mL against MRSA, followed by non-nanoformulated Cin with an MIC of 1,024 μg/mL. This observation is consistent with Badr et al.’s findings (Makimori et al., 2020), which also highlighted the substantial impact of nanoemulsion formulation on Cin’s effectiveness. The inherent hydrophobic nature of essential oils can limit their interaction with bacteria in an aqueous environment, delaying their bactericidal efficacy and potentially reducing bacterial toxicity (Burt and Reinders, 2003; Ultee et al., 2002). By converting this hydrophobic compound into an oil-in-water (O/W) nanoemulsion, we facilitate the emulsifier particles’ fusion with the bacterial cell membrane’s phospholipid bilayer. This mechanism is anticipated to enhance the targeted delivery of active components to their intended sites and prolong the sustained release of these ingredients from the nanoemulsion particles. Additionally, the electrostatic interaction between the charged particles and microbial cell walls intensifies the accumulation of active ingredients at the site of action, thereby amplifying therapeutic outcomes.

In this research, the novel Cin-RHL-NE formulation not only improved Cin’s solubility and stability but also demonstrated superior performance in eradicating both planktonic MRSA and established biofilms. Scanning Electron Microscopy (SEM) analyses revealed that MRSA cells treated with the formulation displayed roughened membranes with particulates and severe wrinkling, leading to rupture, in stark contrast to the untreated control group. Confocal Laser Scanning Microscopy (CLSM) further illuminated cellular abnormalities in MRSA treated with Cin-RHL-NE, revealing significant cytoplasmic leakage and profound cellular deformities. These findings align with prior research, which highlights how nanoemulsions can dramatically enhance antibacterial efficacy. This heightened efficacy results from the increased surface area and substantial presence of emulsifiers that facilitate intimate interaction with the bacterial cell membrane’s phospholipid bilayer, ultimately impairing cell function (Pan et al., 2023).

Our Cin-RHL-NE formulation, therefore, effectively utilizes nanoemulsion technology to harness Cin’s antimicrobial potential while leveraging RHL’s targeting capability to enhance biofilm penetration and disruption. This represents a sophisticated strategy against MRSA infections. Furthermore, the data conclusively show that the Cin-RHL-NE formulation outperforms Cin-NE in its efficacy against biofilms. Guo et al. (2021) used an alternating solvent crystallization and precipitation (ASCP) technique to create protein/polysaccharide/biosurfactant ternary complexes with RHL. Their report indicated that complexes without RHL had significantly larger particle sizes (821 nm) compared to those with RHL (228 nm). This suggests that RHL inhibits particle aggregation, acting similarly to a conventional denaturant. By inducing proteins or polysaccharides to aggregate into compact clusters, RHL fosters a unique structural organization. Since biofilms primarily consist of proteins, polysaccharides, and extracellular DNA, RHL’s ability to modulate protein and polysaccharide aggregation confers a targeting potential.

A principal limitation remains in the as-yet-undefined mechanisms underlying the Cin-RHL-NE’s ability to dismantle MRSA biofilms. Nonetheless, this study posits Cin-RHL-NE as a promising candidate for biofilm eradication, showing potential both *in vitro* and, hypothetically, *in vivo*. Its ability to infiltrate biofilm architecture and dismantle these resilient structures addresses a critical challenge in MRSA biofilm management and may help alleviate antibiotic resistance by exploring alternative therapeutic approaches.

### 4.1 Resource identification initiative

To take part in the Resource Identification Initiative, please use the corresponding catalog number and RRID in your current manuscript. For more information about the project and for steps on how to search for an RRID, please click here.

## Data Availability

The original contributions presented in the study are included in the article/supplementary material, further inquiries can be directed to the corresponding authors.

## References

[ref1] Anjum V. Bagale U. Kadi A. Malinin A. Potoroko I. Alharbi A. H.. (2024). Process optimization of tinospora cordifolia extract-loaded water in oil nanoemulsion developed by ultrasound-assisted homogenization. Molecules 29:1797. doi: 10.3390/molecules29081797 38675617 PMC11052499

[ref2] Arendrup M. C. Prakash A. Meletiadis J. Sharma C. Chowdhary A. (2017). Comparison of eucast and clsi reference microdilution mics of eight antifungal compounds for candida auris and associated tentative epidemiological cutoff values. Antimicrob. Agents Chemother. 61, 10–1128. doi: 10.1128/AAC.00485-17 PMC5444165 28416539

[ref3] Bhattacharya S. (2021). “Central composite design for response surface methodology and its application in pharmacy” in Response surface methodology in engineering science. ed. Kayaroganam P. (Rijeka: IntechOpen).

[ref4] Burt S. A. Reinders R. D. (2003). Antibacterial activity of selected plant essential oils against escherichia coli o157:h7. Lett. Appl. Microbiol. 36, 162–167. doi: 10.1046/j.1472-765x.2003.01285.x 12581376

[ref5] Chalchat J. Valade I. (2000). Chemical composition of leaf oils of Cinnamomum from Madagascar: C-zeylanicum Blume, C-camphora L., C-fragrans Baillon and C-angustifolium. JEOR 12, 537–540. doi: 10.1080/10412905.2000.9712153

[ref6] Chambers H. F. Deleo F. R. (2009). Waves of resistance: *staphylococcus aureus* in the antibiotic era. Nat. Rev. Microbiol. 7, 629–641. doi: 10.1038/nrmicro2200 19680247 PMC2871281

[ref7] Chen J. Li S. Zheng Q. Feng X. Tan W. Feng K.. (2022). Preparation of solid lipid nanoparticles of cinnamaldehyde and determination of sustained release capacity. Nanomaterials 12:4460. doi: 10.3390/nano12244460 36558312 PMC9785162

[ref8] Cruz C. D. Shah S. Tammela P. (2018). Defining conditions for biofilm inhibition and eradication assays for gram-positive clinical reference strains. BMC Microbiol. 18:173. doi: 10.1186/s12866-018-1321-6 30390625 PMC6215609

[ref9] DeLeo F. R. Otto M. Kreiswirth B. N. Chambers H. F. (2010). Community-associated meticillin-resistant *staphylococcus aureus*. Lancet 375, 1557–1568. doi: 10.1016/S0140-6736(09)61999-1 20206987 PMC3511788

[ref10] Donsi F. Annunziata M. Vincensi M. Ferrari G. (2012). Design of nanoemulsion-based delivery systems of natural antimicrobials: effect of the emulsifier. J. Biotechnol. 159, 342–350. doi: 10.1016/j.jbiotec.2011.07.001 21763730

[ref11] Dubinin M. V. Semenova A. A. Ilzorkina A. I. Mikheeva I. B. Yashin V. A. Penkov N. V.. (2020). Effect of betulin and betulonic acid on isolated rat liver mitochondria and liposomes. Biochim. Biophys. Acta Biomembr. 1862:183383. doi: 10.1016/j.bbamem.2020.183383 32522531

[ref12] Feng X. Feng K. Zheng Q. Tan W. Zhong W. Liao C.. (2022). Preparation and characterization of geraniol nanoemulsions and its antibacterial activity. Front. Microbiol. 13:1080300. doi: 10.3389/fmicb.2022.1080300 36523845 PMC9745324

[ref13] Feng J. Sun L. Zhai T. Liang Q. Jiang T. Chen Z. (2023). Preparation of cinnamaldehyde nanoemulsions: formula optimization, antifungal activity, leaf adhesion, and safety assessment. Ind. Crop. Prod. 200:116825:116825. doi: 10.1016/j.indcrop.2023.116825

[ref14] Gambino E. Maione A. Guida M. Albarano L. Carraturo F. Galdiero E.. (2022). Evaluation of the pathogenic-mixed biofilm formation of pseudomonas aeruginosa/staphylococcus aureus and treatment with limonene on three different materials by a dynamic model. Int. J. Environ. Res. Public Health 19:3741. doi: 10.3390/ijerph19063741 35329426 PMC8955688

[ref15] Gu K. Ouyang P. Hong Y. Dai Y. Tang T. He C.. (2022). Geraniol inhibits biofilm formation of methicillin-resistant staphylococcus aureus and increase the therapeutic effect of vancomycin in vivo. Front. Microbiol. 13:960728. doi: 10.3389/fmicb.2022.960728 36147840 PMC9485828

[ref16] Guo Q. Shu X. Hu Y. Su J. Chen S. Decker E. A.. (2021). Formulated protein-polysaccharide-surfactant ternary complexes for co-encapsulation of curcumin and resveratrol: characterization, stability and in vitro digestibility. Food Hydrocoll. 111:106265. doi: 10.1016/j.foodhyd.2020.106265

[ref17] Hassan D. Omolo C. A. Fasiku V. O. Mocktar C. Govender T. (2020). Novel chitosan-based ph-responsive lipid-polymer hybrid nanovesicles (Ola-lphvs) for delivery of vancomycin against methicillin-resistant *staphylococcus aureus* infections. Int. J. Biol. Macromol. 147, 385–398. doi: 10.1016/j.ijbiomac.2020.01.019 31926237

[ref18] He Z. Zeng W. Zhu X. Zhao H. Lu Y. Lu Z. (2017). Influence of surfactin on physical and oxidative stability of microemulsions with docosahexaenoic acid. Colloids Surf. B Biointerfaces 151, 232–239. doi: 10.1016/j.colsurfb.2016.12.026 28013167

[ref19] Inoue D. Kabata T. Ohtani K. Kajino Y. Shirai T. Tsuchiya H. (2017). Inhibition of biofilm formation on iodine-supported titanium implants. Int. Orthop. 41, 1093–1099. doi: 10.1007/s00264-017-3477-3 28386730

[ref20] Jamal M. Ahmad W. Andleeb S. Jalil F. Imran M. Nawaz M. A.. (2018). Bacterial biofilm and associated infections. J. Chin. Med. Assoc. 81, 7–11. doi: 10.1016/j.jcma.2017.07.012 29042186

[ref21] Ji H. Dong K. Yan Z. Ding C. Chen Z. Ren J.. (2016). Bacterial hyaluronidase self-triggered prodrug release for chemo-photothermal synergistic treatment of bacterial infection. Small 12, 6200–6206. doi: 10.1002/smll.201601729 27690183

[ref22] Kavanaugh N. L. Ribbeck K. (2012). Selected antimicrobial essential oils eradicate pseudomonas spp. and *Staphylococcus aureus* biofilms. Appl. Environ. Microbiol. 78, 4057–4061. doi: 10.1128/AEM.07499-11 22467497 PMC3346404

[ref23] Khan A. W. Kotta S. Ansari S. H. Sharma R. K. Ali J. (2015). Self-nanoemulsifying drug delivery system (snedds) of the poorly water-soluble grapefruit flavonoid naringenin: design, characterization, in vitro and in vivo evaluation. Drug Deliv. 22, 552–561. doi: 10.3109/10717544.2013.878003 24512268

[ref24] Lei J. Gao Y. Ma Y. Zhao K. Du F. (2019). Improving the emulsion stability by regulation of dilational rheology properties. Colloids Surf. A Physicochem. Eng. Asp. 583:123906:123906. doi: 10.1016/j.colsurfa.2019.123906

[ref25] Li Y. Jin H. Sun X. Sun J. Liu C. Liu C.. (2018). Physicochemical properties and storage stability of food protein-stabilized nanoemulsions. Nanomaterials 9:25. doi: 10.3390/nano9010025 30585224 PMC6359652

[ref26] Li Q. Liu Q. Wang Z. Zhang X. Ma R. Hu X.. (2023). Biofilm homeostasis interference therapy via (1) o(2) -sensitized hyperthermia and immune microenvironment re-rousing for biofilm-associated infections elimination. Small 19:e2300592. doi: 10.1002/smll.202300592 36850031

[ref27] Lin L. Mao X. Sun Y. Cui H. (2018). Antibacterial mechanism of artemisinin / beta-cyclodextrins against methicillin-resistant *Staphylococcus aureus* (mrsa). Microb. Pathog. 118, 66–73. doi: 10.1016/j.micpath.2018.03.014 29530805

[ref28] Liu Q. Wang J. Wu H. Zong S. Wang N. Wang T.. (2022). Structure and pseudo-ternary phase diagram of water/triton x-100/1-pentanol/cyclohexane microemulsion. J. Mol. Liq. 349:118425.

[ref29] Makimori R. Y. Endo E. H. Makimori J. W. Zanqueta E. B. Ueda-Nakamura T. Leimann F. V.. (2020). Preparation, characterization and antidermatophytic activity of free- and microencapsulated cinnamon essential oil. J. Mycol. Med. 30:100933. doi: 10.1016/j.mycmed.2020.100933 32061515

[ref30] Mohanty S. Mukherji S. (2013). Surfactant aided biodegradation of napls by burkholderia multivorans: comparison between triton x-100 and rhamnolipid jbr-515. Colloids Surf. B Biointerfaces 102, 644–652. doi: 10.1016/j.colsurfb.2012.08.064 23104033

[ref31] Nelson R. (2003). Antibiotic development pipeline runs dry. New drugs to fight resistant organisms are not being developed, experts say. Lancet 362, 1726–1727. doi: 10.1016/s0140-6736(03)14885-4 14655659 PMC7135628

[ref32] Pan Q. Zhou C. Yang Z. Wang C. He Z. Liu Y.. (2023). Preparation and characterization of functionalized chitosan/polyvinyl alcohol composite films incorporated with cinnamon essential oil as an active packaging material. Int. J. Biol. Macromol. 235:123914. doi: 10.1016/j.ijbiomac.2023.123914 36870659

[ref33] Peng Q. Lin F. Ling B. (2020). In vitro activity of biofilm inhibitors in combination with antibacterial drugs against extensively drug-resistant acinetobacter baumannii. Sci. Rep. 10:18097. doi: 10.1038/s41598-020-75218-y 33093606 PMC7581519

[ref34] Qin S. Wen Z. Huang H. Wu W. (2024). Use of novel taurine-chitosan mediated liposomes for enhancing the oral absorption of doxorubicin via the taut transporter. Carbohydr. Polym. 329:121780. doi: 10.1016/j.carbpol.2024.121780 38286550

[ref35] Scheinpflug K. Krylova O. Strahl H. (2017). Measurement of cell membrane fluidity by laurdan gp: fluorescence spectroscopy and microscopy. Methods Mol. Biol. 1520, 159–174. doi: 10.1007/978-1-4939-6634-9_10 27873252

[ref36] Shen S. Zhang T. Yuan Y. Lin S. Xu J. Ye H. (2015). Effects of cinnamaldehyde on escherichia coli and *staphylococcus aureus* membrane. Food Control 47, 196–202. doi: 10.1016/j.foodcont.2014.07.003

[ref37] Singhai M. Malik A. Shahid M. Malik M. A. Goyal R. (2012). A study on device-related infections with special reference to biofilm production and antibiotic resistance. J. Glob. Infect Dis. 4, 193–198. doi: 10.4103/0974-777X.103896 23326076 PMC3543538

[ref38] Su Z. Kong L. Mei J. Li Q. Qian Z. Ma Y.. (2023). Enzymatic bionanocatalysts for combating peri-implant biofilm infections by specific heat-amplified chemodynamic therapy and innate immunomodulation. Drug Resist. Updat. 67:100917. doi: 10.1016/j.drup.2022.100917 36608472

[ref39] Thakur P. Saini N. K. Thakur V. K. Gupta V. K. Saini R. V. Saini A. K. (2021). Rhamnolipid the glycolipid biosurfactant: emerging trends and promising strategies in the field of biotechnology and biomedicine. Microb. Cell Factories 20:1. doi: 10.1186/s12934-020-01497-9 PMC7784359 33397389

[ref40] Topa S. H. Subramoni S. Palombo E. A. Kingshott P. Rice S. A. Blackall L. L. (2018). Cinnamaldehyde disrupts biofilm formation and swarming motility of pseudomonas aeruginosa. Microbiology 164, 1087–1097. doi: 10.1099/mic.0.000692 29993359

[ref41] Tursi S. A. Puligedda R. D. Szabo P. Nicastro L. K. Miller A. L. Qiu C.. (2020). *Salmonella typhimurium* biofilm disruption by a human antibody that binds a pan-amyloid epitope on curli. Nat. Commun. 11:1007. doi: 10.1038/s41467-020-14685-3 32081907 PMC7035420

[ref42] Ultee A. Bennik M. H. Moezelaar R. (2002). The phenolic hydroxyl group of carvacrol is essential for action against the food-borne pathogen bacillus cereus. Appl. Environ. Microbiol. 68, 1561–1568. doi: 10.1128/AEM.68.4.1561-1568.2002 11916669 PMC123826

[ref43] Ye J. Hou F. Chen G. Zhong T. Xue J. Yu F.. (2023). Novel copper-containing ferrite nanoparticles exert lethality to mrsa by disrupting mrsa cell membrane permeability, depleting intracellular iron ions, and upregulating ros levels. Front. Microbiol. 14:1023036. doi: 10.3389/fmicb.2023.1023036 36846790 PMC9947852

[ref44] Ying Yang C. M. M. E. (2012). Fabrication of ultrafine edible emulsions: comparison of high-energy and low-energy homogenization methods. Food Hydrocoll. 29, 398–406. doi: 10.1016/j.foodhyd.2012.04.009

[ref45] Yuan Q. Feng W. Wang Y. Wang Q. Mou N. Xiong L.. (2022). Luteolin attenuates the pathogenesis of *Staphylococcus aureus* by interfering with the agr system. Microb. Pathog. 165:105496. doi: 10.1016/j.micpath.2022.105496 35331848

[ref46] Yuan Gao Q. L. Z. W. (2021). Cinnamaldehyde nanoemulsions; physical stability, antibacterial properties/mechanisms, and biosafety. J. Food Meas. Charact. doi: 10.1007/s11694-021-01110-6

[ref47] Zhang L. Wen B. Bao M. Cheng Y. Mahmood T. Yang W.. (2021). Andrographolide sulfonate is a promising treatment to combat methicillin-resistant staphylococcus aureus and its biofilms. Front. Pharmacol. 12:720685. doi: 10.3389/fphar.2021.720685 34603031 PMC8481920

[ref48] Zhang H. Zhou W. Zhang W. Yang A. Liu Y. Jiang Y.. (2014). Inhibitory effects of citral, cinnamaldehyde, and tea polyphenols on mixed biofilm formation by foodborne staphylococcus aureus and *Salmonella enteritidis*. J. Food Prot. 77, 927–933. doi: 10.4315/0362-028X.JFP-13-497 24853514

[ref49] Zuberi A. Ahmad N. Khan A. U. (2017a). Crispri induced suppression of fimbriae gene (fimh) of a uropathogenic escherichia coli: an approach to inhibit microbial biofilms. Front. Immunol. 8:1552. doi: 10.3389/fimmu.2017.01552 29181009 PMC5694031

[ref50] Zuberi A. Azam M. W. Khan A. U. (2022). Crispr interference (crispri) mediated suppression of ompr gene in *E. coli*: an alternative approach to inhibit biofilm. Curr. Microbiol. 79:78. doi: 10.1007/s00284-021-02760-x 35091832

[ref51] Zuberi A. Misba L. Khan A. U. (2017b). Crispr interference (crispri) inhibition of luxs gene expression in *E. coli*: an approach to inhibit biofilm. Front. Cell. Infect. Microbiol. 7:214. doi: 10.3389/fcimb.2017.00214 28603699 PMC5445563

